# Dialysis modality and cognitive outcomes in chronic kidney disease: a systematic review and meta-analysis

**DOI:** 10.1007/s10157-025-02798-2

**Published:** 2025-12-01

**Authors:** Ali Malik, Hamid Reza Khademi Mansour, Sukruth Pradeep Kundur, Aryan Hunjan, Rumail Zaheer

**Affiliations:** 1 Faculty of Life Sciences and Medicine, King’s College London, London, UK; 2 George’s University Hospitals NHS Foundation Trust, London, UK

**Keywords:** Chronic kidney disease, Haemodialysis, Peritoneal dialysis

## Abstract

**Background:**

Cognitive impairment is a prevalent comorbidity in patients with chronic kidney disease (CKD). While hemodialysis (HD) and peritoneal dialysis (PD) are established renal replacement therapies, their relative effects on cognitive outcomes remain unclear. This meta-analysis compared cognitive outcomes between HD and PD in CKD patients.

**Methods:**

The protocol was prospectively registered on PROSPERO (CRD42024602533). PubMed, CENTRAL, Embase, Medline, Web of Science, PsychInfo, and CINAHL were searched from January 2000 to January 2025. Eligible studies included cohort studies of adult patients undergoing HD versus PD. Primary outcomes were cognitive function and dementia incidence. A random-effects meta-analysis model was used. Risk of bias was assessed using ROBINS-I, and evidence quality was evaluated using GRADE. Methodological rigor was benchmarked against previous reviews using AMSTAR 2.0.

**Results:**

The search identified 1489 studies, of which 26, involving 326,216 patients, were included. There was a statistically significant difference in overall cognitive function between HD and PD (SMD: −0.46; 95% CI: −0.62 to –0.29; *p* < 0.00001; I^2^ = 49%), and dementia incidence (OR: 1.68; 95% CI: 1.25 to 2.25; *p* = 0.0006; I^2^ = 94%). Subgroup and qualitative analyses suggested PD offers advantages in executive function, verbal memory, and cognitive stability.

**Conclusions:**

Quantitative analyses revealed significant evidence, and qualitative trends suggest PD may be associated with better cognitive outcomes in select domains. These findings underscore the need to individualize dialysis modality decisions based on cognitive risk profiles and conduct further standardized research.

**Supplementary Information:**

The online version contains supplementary material available at 10.1007/s10157-025-02798-2.

## Introduction

Cognitive impairment (CI) is a significant contributor to disability, commonly assessed and quantified using validated neurocognitive assessment tools such as the Mini-Mental State Examination (MMSE) and the Montreal Cognitive Assessment (MoCA) [1, 2]. CI encompasses deficits in executive functioning, memory, attention, and visuospatial skills, significantly impacting patients' quality of life and increasing the risk of progression towards dementia [3, 4]. Notably, CI is increasingly recognised as a prevalent comorbidity among individuals with chronic kidney disease (CKD), particularly those with advanced stages or suboptimal disease management [5].

The pathophysiology underlying the relationship between CKD and CI remains incompletely understood; however, several hypotheses have been proposed. Accumulation of uremic toxins is considered a key pathological factor contributing to cognitive deterioration in CKD patients. These toxins adversely affect neural progenitor cells, impairing their differentiation and survival, which diminishes neuronal repair and neurogenesis capabilities [6]. Additionally, uremic toxins can cause structural and functional changes in cerebral vasculature, including endothelial dysfunction, impaired cerebral autoregulation, and increased blood–brain barrier permeability [7]. Alterations in the glymphatic and lymphatic drainage systems further exacerbate cerebral toxin accumulation, potentiating neuronal injury [8]. Moreover, disturbances in monoaminergic neurotransmitter systems—specifically involving dopamine, serotonin, and norepinephrine—have also been implicated in cognitive dysfunction among CKD patients, potentially contributing to deficits in attention, executive function, and mood regulation [9].

The prevalence of CI among CKD patients is substantial, estimated to range between 33 and 46%, highlighting a critical need to identify effective therapeutic strategies to mitigate cognitive decline within this patient population [10]. Renal replacement therapy (RRT), primarily through hemodialysis (HD) and peritoneal dialysis (PD), remains the gold standard of treatment to manage CKD and reduce uremic toxin load. HD is typically used in patients requiring rapid removal of toxins, severe electrolyte imbalances, or those with limited peritoneal membrane function. Conversely, PD offers greater hemodynamic stability and continuous toxin clearance, potentially yielding differing impacts on cognitive functions compared to HD [11, 12].

Despite the widespread use of these dialysis modalities, uncertainty persists regarding their comparative effectiveness in preserving cognitive functions in patients undergoing dialysis, as existing literature reports conflicting findings [13, 14].

Two previously published systematic reviews exploring the comparative effects of PD and HD on cognitive outcomes suggest that PD may provide superior benefits in preserving cognitive functions [15, 16]. However, these reviews are limited by methodological inadequacies, such as small sample sizes, variability in cognitive assessment measures, and outdated literature searches, with the most recent publication occurring in 2021. Given the rapid accumulation of new data in recent years, an updated systematic review is warranted to incorporate recent evidence and address methodological weaknesses observed in prior reviews.

This systematic review aims to evaluate and compare the effectiveness of PD and HD in mitigating cognitive decline among CKD patients. Through a comprehensive systematic review and meta-analysis, this review seeks to quantify differences in cognitive outcomes between dialysis modalities, identify specific cognitive domains predominantly affected by each treatment, and explore patient-specific and dialysis-related factors influencing cognitive trajectories. Ultimately, findings from this review will provide robust, evidence-based insights to inform clinical decision-making and refine dialysis management strategies, optimising patient outcomes in cognitive health among those receiving renal replacement therapy.

## Methodology

The protocol for this systematic review and meta-analysis was prospectively registered with the International Prospective Register of Systematic Reviews (PROSPERO; Registration number: CRD42024602533) [17]. The review was conducted in strict accordance with the Preferred Reporting Items for Systematic Reviews and Meta-Analyses (PRISMA) guidelines to ensure methodological rigor, transparency, and reproducibility [18]. Each domain of the PRISMA checklist is cross-referenced in Online Resource 1.

### Search strategy

A comprehensive search was undertaken across seven electronic databases: PubMed, CENTRAL, Embase, Medline, Web of Science, PsychInfo, and CINAHL. Searches were conducted without restrictions on language or study design. However, a publication date restriction was set from January 2000 to January 2025. This timeframe was specifically chosen due to the limited recognition of CI as a significant comorbidity associated with CKD and dialysis modalities in literature prior to 2000, thereby ensuring contemporary clinical relevance. The database searches combined text words and Medical Subject Headings (MeSH) terms. A sample search strategy for PubMed is as follows: (chronic kidney disease OR CKD OR CKD [MeSH] OR end-stage renal disease OR ESRD OR ESRD [MeSH]) AND (cognit* OR cognition [MeSH] OR executive function OR executive dysfunction OR dementia OR delirium OR amnestic OR mental status OR neuropsych* OR memory) AND (hemodialysis OR haemodialysis) AND (peritoneal dialysis).

Complete details of search strategies and individual database search results are provided in Online Resource 2.

### Study selection

Search results from all databases were imported into Rayyan [19], and duplicate entries were automatically identified and removed. Following deduplication, two authors (A.M. & S.P.K.) independently screened the titles and abstracts of all identified records to determine potential eligibility based on predefined inclusion and exclusion criteria. Subsequently, full-text versions of potentially eligible studies were retrieved and independently assessed for final eligibility by the same two reviewers. Any disagreements at any stage of the selection process were resolved through consensus discussions or, if necessary, consultation with a third reviewer.

Eligible studies included non-randomized comparative studies and randomised controlled trials (RCTs) enrolling adult patients (≥ 18 years) diagnosed with CKD and receiving either HD or PD. Studies were excluded if they: (1) involved pediatric populations (< 18 years), (2) did not explicitly report cognitive outcomes of interest, (3) did not directly compare PD with HD (e.g., included only one modality without direct comparison, or compared dialysis modalities against non-dialysis treatments), or (4) were unavailable in full-text form despite efforts to obtain the document.

Risk of bias assessment was systematically performed using the Cochrane Risk of Bias tool (RoB 2.0) [20] for RCTs and the Risk of Bias in Non-randomized Studies of Interventions (ROBINS-I) tool [21] for non-randomized comparative studies. The screening and selection process was transparently documented using a PRISMA flow diagram, clearly indicating reasons for exclusion at every stage. The quality and methodological robustness of prior systematic reviews addressing similar research questions were critically appraised using the AMSTAR 2.0 tool [22]. The overall quality of the evidence generated by the included studies was graded using the Grading of Recommendations Assessment, Development, and Evaluation (GRADE) framework [23].

### Data extraction

A standardised and pre-designed data extraction form was developed to capture variables relevant to the primary and secondary outcomes of interest. Two reviewers (A.M. & S.P.K.) independently extracted data from the included studies, systematically recording study-level characteristics such as author names, year of publication, country of origin, study design, study setting, funding sources, duration of follow-up, outcomes reported, primary findings, and detailed inclusion/exclusion criteria.

Patient characteristics collected included dialysis modality (HD or PD), age, sex distribution, educational background, hypertension status, diabetes mellitus, smoking status, alcohol intake, socioeconomic status (e.g., income, work status), and other relevant demographic or clinical factors identified by included studies. Intervention characteristics, including dialysis regimen specifics (e.g., frequency, duration per session, duration on dialysis), were thoroughly recorded.

Cognitive outcome measures encompassed results from neuropsychological assessments (e.g., MMSE, MoCA, domain-specific tests), neuroimaging findings (when available), and psychological or psychiatric evaluations. Statistical parameters extracted included details on analytic methods, effect sizes, standard deviations, confidence intervals, heterogeneity indicators, and assessments for publication bias. Whenever data was incomplete or unclear, corresponding study authors were contacted for clarification. If missing data could not be obtained, its nature and potential implications for study results were transparently reported.

### Data analysis

All statistical analyses were conducted using Review Manager software (RevMan Version 5.4.1; Copenhagen: The Nordic Cochrane Centre, The Cochrane Collaboration, 2020) [24]. Outcome data, specifically mean cognitive scores and standard deviations, were pooled using a random-effects model to accommodate anticipated heterogeneity across included studies. Results were expressed as weighted mean differences with corresponding 95% confidence intervals (CIs), graphically represented via forest plots.

Statistical significance was set at an alpha level of *p* < 0.05. Heterogeneity among included studies was quantitatively evaluated using the I^2^ statistic according to guidelines recommended by the Cochrane Handbook for Systematic Reviews of Interventions [25]. Where considerable heterogeneity (I^2^ ≥ 50%) was identified, sensitivity analyses or subgroup analyses were considered. In instances where quantitative pooling of data was not feasible or advisable due to high heterogeneity or insufficient data, a narrative synthesis approach was undertaken.

## Results

### Study and patient characteristics

**Fig. 1:**
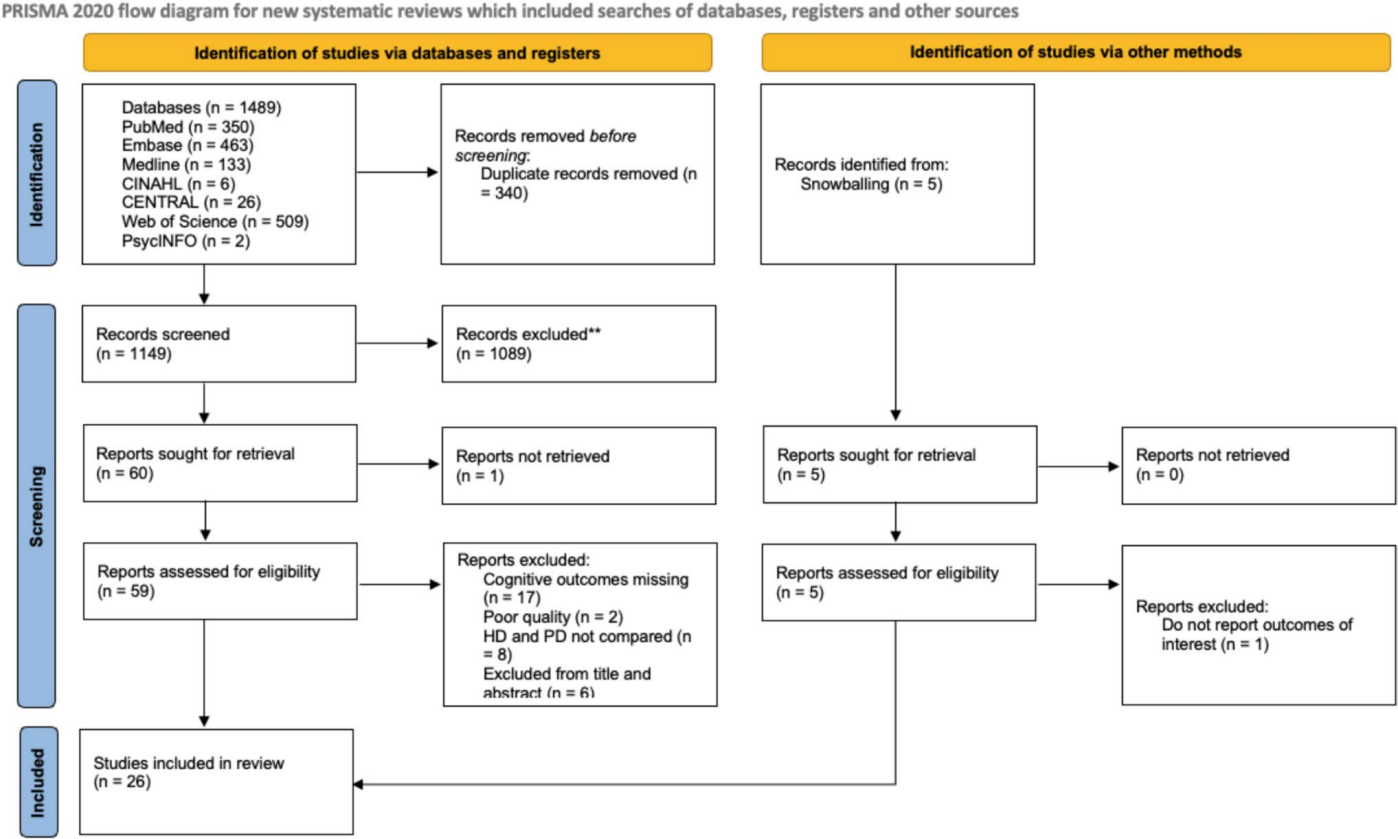
PRISMA flow diagram illustrating the stages of article selection for this systematic review, from initial database search to final study inclusion, with numbers at each stage (created using the official PRISMA 2020 template in Microsoft PowerPoint)

The database search identified a total of 1489 articles. Following rigorous screening and the application of predetermined exclusion criteria, 26 studies were deemed eligible and included in this review [26–51]. A total of 37 studies were excluded during the full-text review, primarily due to a lack of cognitive outcome reporting or the absence of direct comparisons between HD and PD. Five additional studies were excluded during data extraction: two studies provided insufficient cognitive outcome data for analysis, one was excluded due to language barriers (non-English), and two other had severe methodological limitations, including insufficient representation of PD patients (only one patient versus nine on HD) and a serious risk of bias as indicated by the ROBINS-I tool. The full screening and selection process is illustrated using a PRISMA flow diagram in Fig. 1. These included studies encompassed 326,216 participants (231,093 receiving HD, 24,614 receiving PD, and 70,509 serving as controls or undergoing alternative dialysis methods). Individual study sample sizes ranged widely from 30 to 145,868 participants. Of the 27 included studies, 20 (74.1%) employed cross-sectional designs, four (14.8%) were retrospective cohort studies, two (7.4%) were longitudinal studies, one (3.7%) was a prospective observational study, one (3.7%) was a pilot cohort study, and one combined cross-sectional and longitudinal methods. Notably, no RCTs were identified. Eighteen studies explicitly provided financial disclosures, with 16 reporting external funding sources. Key study characteristics are displayed in Table 1, and full study characteristics in Online Resource 3. Cognitive assessments utilised across studies varied considerably; five studies employed the Montreal Cognitive Assessment (MoCA), seven utilized the Mini-Mental State Examination (MMSE), seven applied the Trail Making Test (TMT), and three studies assessed dementia incidence. Additional cognitive assessment tools included the Mental Component Summary (MCS) of the Kidney Disease Quality of Life Short Form (KDQOL-SF v1.3), Brief Cognitive State Examination (BCSE), Symbol Digit Modalities Test (SDMT), Gray Matter Ratio (GMR), European Organization for Research and Treatment of Cancer Quality of Life Questionnaire (QLQ-C30), Cognitive Reserve Index Questionnaire (CRIq), Telephone Modified Mini-Mental State Examination (TMSE), Rey Auditory Verbal Learning Test (RAVLT), and the Modified Mini-Mental State Examination (3MS). Key baseline patient characteristics are presented in Table 2, with more detailed characteristics presented in Online Resource 4. Included studies demonstrated broadly similar demographic profiles between HD and PD cohorts, with mean ages and gender distributions comparable across studies. However, educational levels varied, as studies reported educational attainment differently, including total years of education and highest qualification achieved. Common comorbidities such as hypertension and diabetes were frequently reported, though their prevalence varied among studies. Reporting of smoking and alcohol use was inconsistent across studies. While demographic and clinical characteristics were comparable between HD and PD groups, heterogeneity persisted in age, gender distribution, education, and comorbidity profiles.

**Table 1:** Summary of the characteristics of this review’s included studies including study design, sample size, outcomes assessed, and key findings

Study (author/year)	Study Design	N Total	N HD	N PD	Follow up time	Main outcomes	Key findings
A C Aloui 2021	Cross-sectional study	91	71	20	N/A	KDQOL SF v1.3, MCS	PD: better PCS & cognition; HD: higher burden
B S Park 2020	Cross-sectional study	40	20	20	N/A	VBM MRI; CERAD-K battery (MMSE, verbal fluency, TMT-B, Stroop)	Significant cognitive decline in PD, no statistical difference in HD
C Zhang 2024	cross-sectional study	101 (49 healthy)	25	27	N/A	MMSE, MoCA; rs-fMRI (fALFF, ReHo)	Brain activity differed between groups; no cognition difference
D Neumann 2018	Longitudinal study	271	163	108	1 year	TMT-B, d2-Test, KDQOL-CF	PD showed better cognition; both groups improved over time
F Wolfgram 2015	Retrospective cohort study	121,623	112,960	8663	Up to 3.75 years	Dementia incidence (ESRD cohort)	PD had lower dementia risk than HD (3.9% vs 7.3%)
F XIao 2024	Cross-sectional study	94 (42 healthy)	25	27	N/A	Brain gray matter (MRI); MMSE, MoCA	Both HD/PD had atrophy vs controls; HD worse in some areas
H C Pi 2016	Cross-sectional study	120 (30 ND)	30	60	N/A	3MS, TMT A/B, RBANS; MRI (WMH, atrophy)	HD had HD patients had more severe neuroimaging abnormalities.severe neuroimaging abnormalities
H Hung 2017	Retrospective cohort study	145,868 (72,934 matched controls)	63,372	9562	4.24 years HD; 3.23 years PD	Dementia incidence (AD, VaD, UnD)	Both HD and PD ↑ dementia risk vs controls (HD aHR 1.64; PD aHR 2.21)
H Ozcan 2015	Cross-sectional study	181 (69 KT)	54	58	N/A	BCSE (cognition); HADS (mood)	Cognitive/mood impairment: HD > PD > transplant
J Radic 2011	Cross-sectional study	42	22	20	N/A	SDMT, CRD-series	No HD–PD difference; higher labs linked to better cognition
K Griva 2003	Cross-sectional study	145	77	68	N/A	TMT; neuropsych testing pre- and post-dialysis	HD improved post-dialysis; PD showed minimal change
K Lambert 2017	cross-sectional study	155	54	25	N/A	MoCA	CI common in dialysis; HD highest prevalence (56%), PD lower (48%)
K Tsuruya 2024	Cross-sectional and longitudinal observational study	107	34	73	Longitudinal: 2 years	Brain atrophy (MRI gray matter ratio)	PD had faster GMR decline over 2 years vs HD
Kalirao 2010	Cross-sectional study	490	338	51	N/A	3MS, HVLRT-R, Color Trails 1 & 2	PD worse memory; HD worse executive function; CI common in both
M Majkowicz 2000	Cross-sectional study	87	65	22	N/A	EORTC QLQ-C30, Cantrill ladder, HADS	HD patients had lower QoL across domains vs controls
M Robinski 2017	cross-sectional study	482	241	241	6–24 months	TS, SDM, TMT, psychological/physical measures	PD patients had higher satisfaction, autonomy, and cognition
O Iyasere 2016	prospective cohort study	102	41	25	4–24 months	MoCA, MacCAT-T	HD declined faster in executive function than PD
P Giuseppe 2024	Cross-sectional study	99 (33 Healthy)	33	33	N/A	Cognitive Reserve Index Questionnaire (CRI-q)	HD had lower cognitive reserve than PD and controls
P Sithinamsuwan 2005	Cross-sectional study	90	60	30	N/A	TMSE, TDI	Dementia more prevalent in HD (8.3%) vs PD (3.3%)
S George 2013	Pilot cohort study	80	59	21	12 months	DST, verbal fluency, digit span, Trails	PD patients declined faster cognitively than HD
S jung 2013	cross-sectional study	56	29	27	N/A	K-MMSE, K-BDI	PD had higher cognition and lower depression than HD
S Lai 2016	cross-sectional study	99	15	16	N/A	EEG; NPZ5 cognition; MMPI-2, SAT-P	CKD & dialysis pts had marked cognitive/psychological impairment
T Hiramtsu 2020	Prospective, observational study	75	45	30	24 months	SF-36, MMSE, CES-D	PD improved QoL; HD declined in MMSE & depression scores
Tilki 2004	cross-sectional study	52	25	17	N/A	P300 latency; MMSE	HD had longer P300 latency than PD and controls
Williams 2004	cross-sectional study	30	20	10	N/A	Stroop, K-BIT, BDI-II	CAPD stable; HD had memory impairments post-dialysis
Y T Lin 2015	Retrospective cohort study	55,264	52,332	3292	N/A	Dementia diagnosis (registry)	Dementia incidence lower in PD than HD (146 vs 178/10 k py)

**Table 2:** Summary of the baseline characteristics of included patients in this reviews’ included studies

Study	Modality	Age (Median, IQR/SD)	Female (%)	HTN (yes, %)	Diabetes (yes, %)	Smoking (yes, %)	Alcohol (yes, %)
A C Aloui 2021	HD	50.0 [35.5; 60.0]	52.1	42.30	9.9	16.9	2.8
	PD	57.5 [42.0; 63.0]	40	65	10	20	5
B S Park 2020	HD	64.85 (7.199)*	60	–	–	–	–
	PD	60.95 (5.414)*	50	–	–	–	–
C Zhang 2024	HD	61.00 ± 11.47	60	88	80	–	–
	PD	54.63 ± 10.27	48.15	92.59	44.44	–	–
D Neumann 2018	HD	57.0 (15.0)	27.6	–	–	–	–
	PD	56.0 (14.7)	34.3	–	–	–	–
F Wolfgram 2015	HD	64.1 ± 14.4	45.5	86.3	50	6.6	–
	PD	62.4 ± 15.9	44.9	85.8	48.9	6.8	–
F XIao 2024	HD	61.0 ± 11.47	40	88	80	–	Excluded (criteria)
	PD	54.63 ± 10.27	51.85	92.59	44.44	–	Excluded (criteria)
H C Pi 2016	HD	56.5 ± 11.8	36.7	–	33.3	–	–
	PD	57.7 ± 7.8	55	–	45	–	–
H Hung 2017	HD	61.2 (13.9)	51.1	88.8	44.6	–	–
	PD	52.9 (15.0)	53.6	89	37.3	–	–
H Ozcan 2015	HD	51.1 ± 12.5	–	–	–	–	–
	PD	51.33 ± 14.4	–	–	–	–	–
	KT	50.19 ± 16.5	–	–	–	–	–
J Radic 2011	HD	49.59 ± 11.64	45.5	–	–	–	–
	PD	51.10 ± 10.66	25	–	–	–	–
K Grava 2003	HD	48.22 ± 14.92	42.9	94.8	7.8	–	–
	PD	52.26 ± 13.26	26.5	88.2	27.9	–	–
K Lambert 2017	PRE	70 (63–76)	54.20%	–	–	–	–
	PD	70 (63–81)	48.00%	–	35.00%	–	–
	HD	72 (58–77)	33.30%	–	51.10%	–	–
	KT	58.5 (49–66)	38.50%	–	28.60%	–	–
K Tsuruya 2024	HD	64 [57–70]	26.40%	Not Reported	35.30%	11.7	38.2
	PD	61 [53–68]	34.20%	Not Reported	39.70%	16.4	52.1
Kalirao 2010	PD	57.5 ± 14.8	33.3	–	41.2	–	–
	HD	71.2 ± 9.5	45.9	–	46.8	–	–
M Majkowicz 2000	HD						
	PD						
M Robinski 2017	HD (PSM)	59.8 ± 15.9	34.4	–	–	–	–
	PD (PSM)	58.8 ± 16.0	27.8	–	–	–	–
O Iyasere 2016	HD	68.9	29.3	–			
	46.3	–	–				
	PD	72.8	24	–	44	–	–
P Giuseppe 2024	HD	65.2 ± 3.4	42	–	18	33	–
	PD	61 ± 5.2	45	–	9	24	–
P Sithinamsuwan 2005	HD	53.67 ± 15.84	45	95	20	–	–
	PD	55.67 ± 14.18	30	83.3	33.3	–	–
S George 2013	HD (PFO)	63.0 ± 11.5	41.7	50	16.7	–	–
	HD (no PFO)	56.7 ± 16.4	28.6	45.7	28.6	–	–
	PD (PFO)	70.0 ± 2.4	60	80	20	–	–
	PD (no PFO)	59.4 ± 15.2	31.8	31.8	68.1	–	–
S jung 2013	HD	55.8 ± 8.7	55.2	–	41.4	20.7	–
	PD	52.4 ± 11.6	48.1	–	33.3	22.2	–
S Lai 2016	HD	53.0 ± 14.1	60	–	–	–	–
	PD	67.0 ± 10.7	37.5	–	–	–	–
T Hiramtsu 2020	HD	66.6 ± 8.4	33.3	–	29.60%	–	–
	PD	63.1 ± 11.0	28.9	–	28.90%	–	–
Tilki 2004	HD	37.3 ± 2.7	48	68%	–	–	–
	PD	44.2 ± 3.9	58.8	65%	–	–	–
Williams 2004	HD	54.6 ± 2.9	50	20	30%	–	–
	PD	45.1 ± 4.8	50	20	50%	–	–
Y T Lin 2015	HD	Mean: 60.4 ± 10.3	52.3	58.20%	47.70%	–	0.60%
	PD	Mean: 59.7 ± 10.5	55.5	64.40%	41.40%	–	0.30%
	PD	58.35 ± 11.07	63.3	27.33	37.33	–	–

### Quantitative analysis

#### Cognitive function

Eighteen studies provided sufficient data on cognitive function to allow pooled meta-analysis. We conducted sub-group meta-analysis based on the four cognitive domains tested by studies: global cognitive function, executive function, processing speed, and memory scores.

**Fig. 2:**
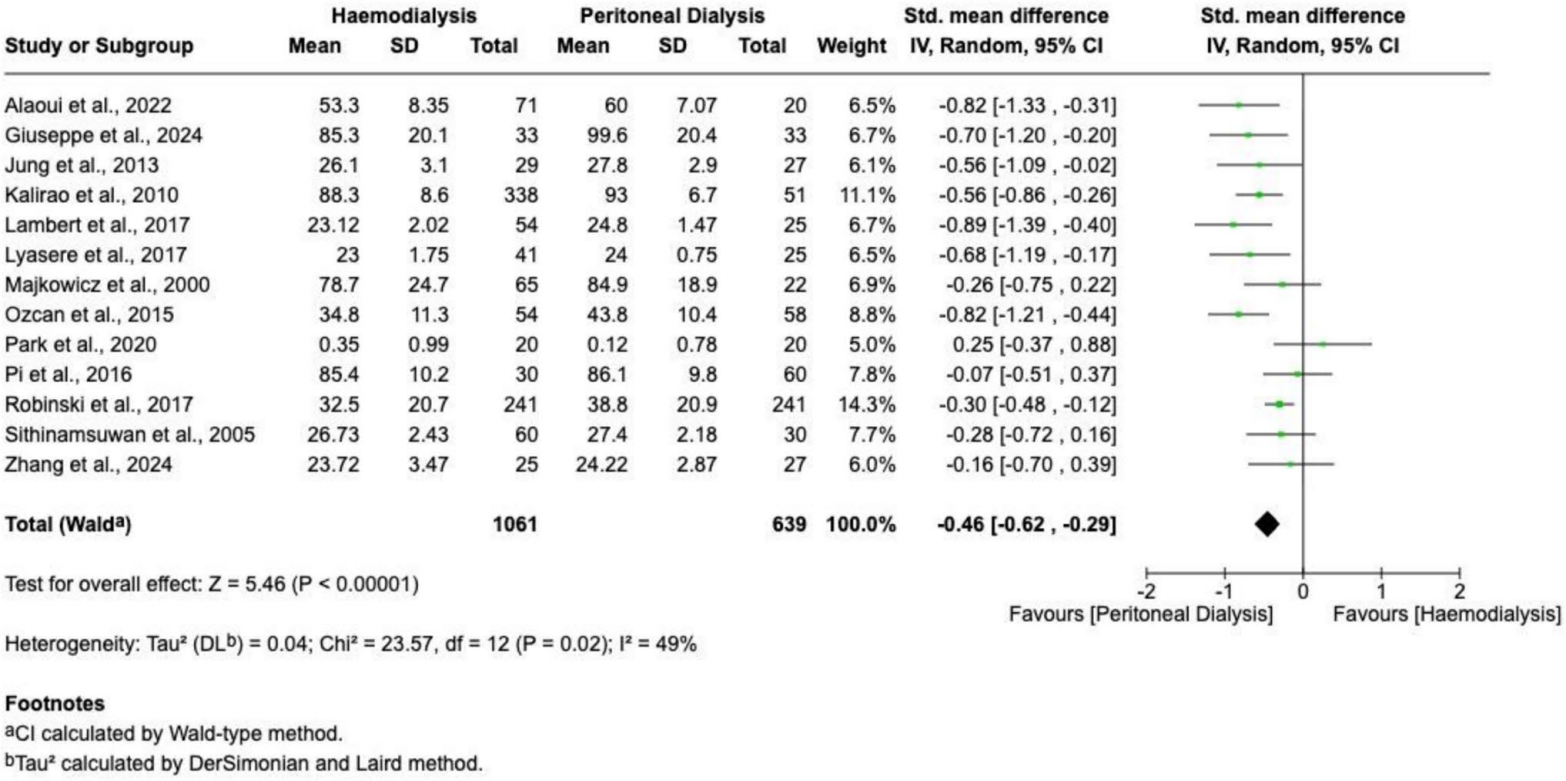
A forest plot visualizing a meta-analysis comparing global cognitive function test scores (generated using RevMan)

Global cognitive function, reported by thirteen studies, revealed significantly improved overall cognitive performance in patients receiving PD (SMD =  − 0.46; 95% CI − 0.62 to − 0.29; *P* < 0.00001; I^2^ = 49%). A forest plot illustrating these results is provided in Fig. 2.

**Fig. 3:**
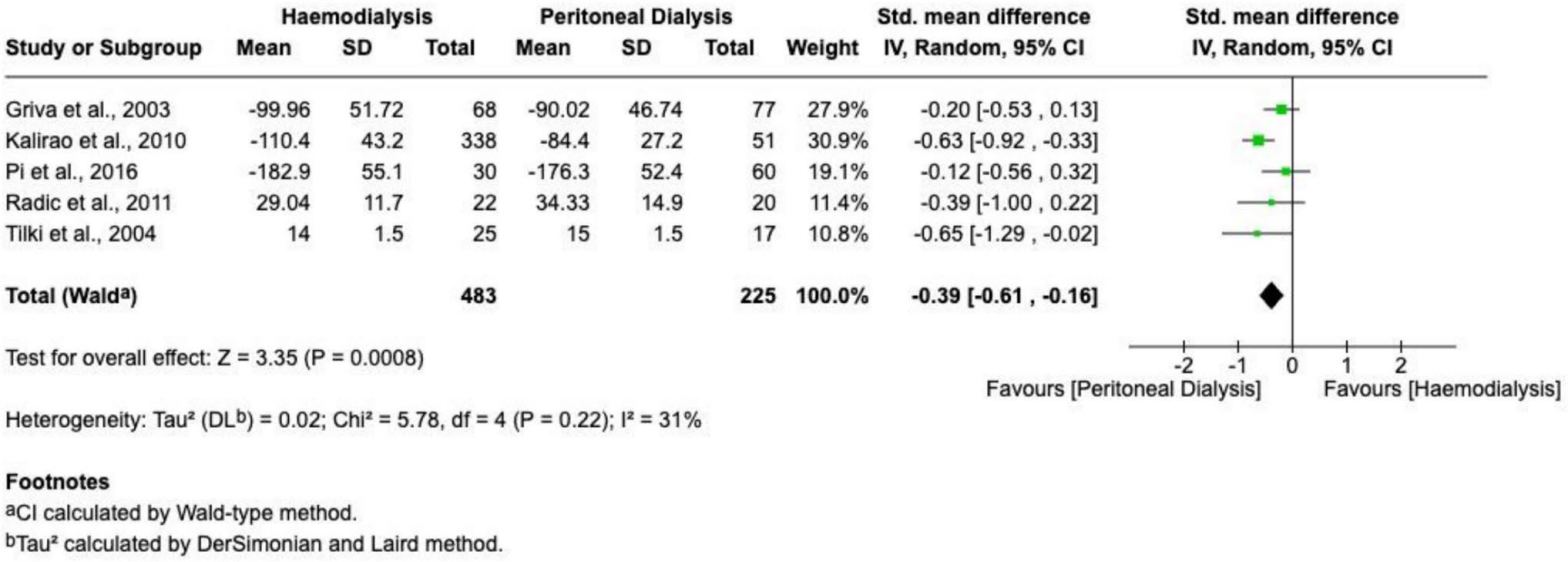
A forest plot visualizing a meta-analysis comparing executive function test scores (generated using RevMan)

Executive function, assessed across five studies, also favoured PD, with significantly better scores compared to HD (SMD =  − 0.39; 95% CI − 0.61 to − 0.16; *P* = 0.0008; I^2^ = 31%). A forest plot illustrating these results is provided in Fig. 3.

**Fig. 4:**
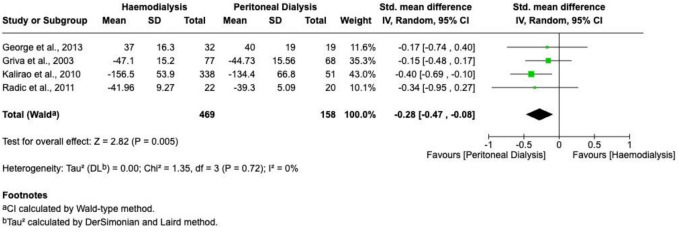
A forest plot visualizing a meta-analysis comparing processing speed test scores (generated using RevMan)

Processing speed was reported by four studies. Pooled analysis demonstrated significantly faster processing speeds among patients undergoing peritoneal dialysis (PD) compared to haemodialysis (HD) (SMD =  − 0.28; 95% CI − 0.47 to − 0.08; *P* = 0.005; I^2^ = 0%). A forest plot illustrating these results is provided in Fig. 4.

**Fig. 5:**
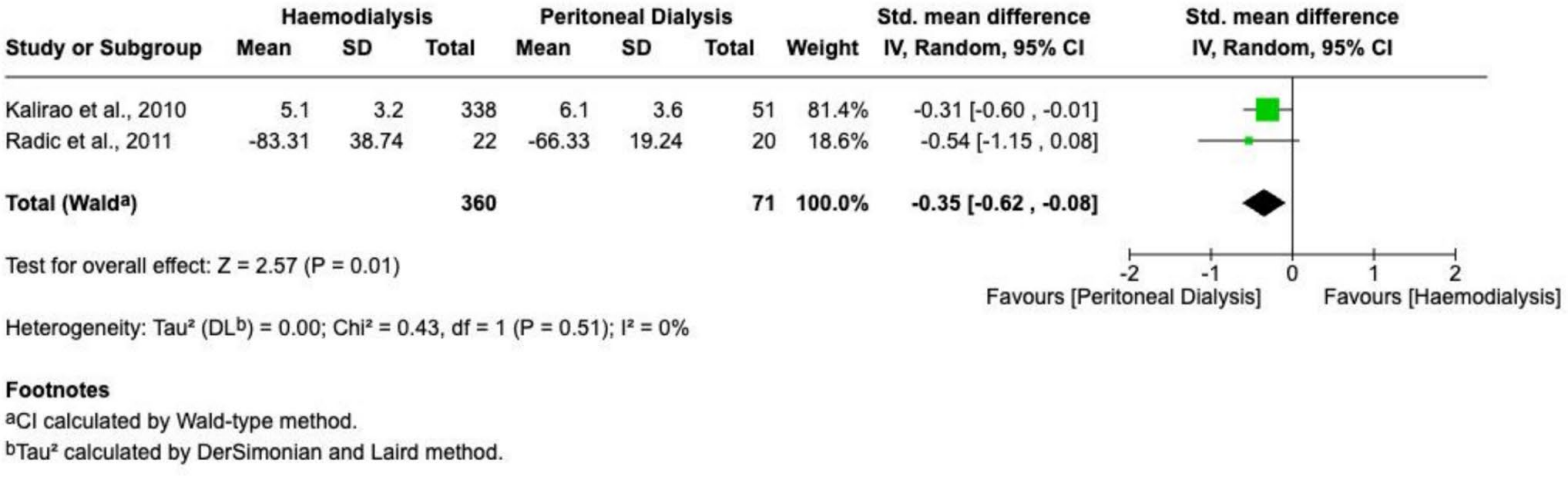
A forest plot visualizing a meta-analysis comparing memory test scores (generated using RevMan)

Memory scores were reported by two studies. The pooled evidence similarly showed significantly better memory performance in PD patients (SMD =  − 0.35; 95% CI − 0.62 to − 0.06; *P* = 0.01; I^2^ = 0%). A forest plot illustrating these results is provided in Fig. 5.

### Incidence of dementia

**Fig. 6:**
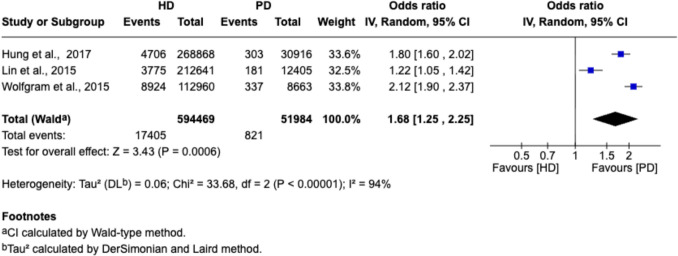
A forest plot visualizing a meta-analysis comparing incidence of dementia across studies comparing this outcome (generated using RevMan)

Incidence of dementia was assessed in three large cohort studies. Pooled analysis demonstrated a significantly lower risk of dementia among PD patients compared to HD patients (OR = 1.68; 95% CI 1.25 to 2.25; *P* = 0.0006; I^2^ = 94%). A forest plot illustrating these results is provided in Fig. 6.

### Qualitative analysis

#### General cognitive trends

Across the included studies, a general trend emerged suggesting superior cognitive outcomes in several domains, specifically executive function, verbal memory, and overall cognitive stability, for patients undergoing PD compared to those on HD. Multiple studies consistently reported enhanced autonomy, slower trajectories of cognitive decline, and reduced dementia risks in PD patients (Neumann et al., 2018; Kalirao et al., 2011; Robinski et al., 2017). Conversely, several studies noted cognitive advantages favoring HD patients, particularly in visuospatial functioning and short-term cognitive recovery immediately following dialysis sessions (George et al., 2013; Lai et al., 2016). Nonetheless, a significant number of studies found no statistically significant differences between the two dialysis modalities, indicating that cognitive outcomes might instead be substantially influenced by patient-specific factors, including age, dialysis duration, and comorbidities (Radic et al., 2011; Sithinamsuwan et al., 2005). Additionally, marked variability in cognitive assessment tools, study designs, and geographic contexts contributed substantially to the observed heterogeneity of results.

### Insights from neuroimaging studies

Several studies incorporated advanced methodologies, such as neuroimaging, offering nuanced insights beyond conventional cognitive assessments. For instance, Zhang et al. (2024) reported comparable results from standardized cognitive testing between HD and PD groups. However, magnetic resonance imaging (MRI) revealed more pronounced structural disruptions in HD patients' frontal and parietal cortical regions, potentially underlying subtle cognitive deficits not detectable through standard neuropsychological tests. Similarly, Xiao et al. (2024) identified more extensive reductions in gray matter volume within memory- and executive-function-related regions, particularly the hippocampus and prefrontal cortex, in HD patients. These findings correlated significantly with poorer cognitive test outcomes. Conversely, Tsuraya et al. (2023) described a faster progression of gray matter atrophy in PD patients over two years. However, both HD and PD groups experienced notable cognitive declines, with atrophy strongly correlating with impaired cognitive performance, specifically on the TMT.

### Longitudinal cognitive changes

Longitudinal studies offered valuable data on the trajectories of cognitive decline associated with dialysis modality over extended follow-up periods. Lyasere et al. (2017) documented a significantly faster deterioration in executive functions and verbal fluency among HD patients compared to their PD counterparts over two years. Conversely, Williams et al. (2004) reported stability in attention and memory domains among PD patients. In contrast, HD patients exhibited marked cognitive impairment at approximately 67 h post-dialysis, suggesting acute, dialysis-related cognitive fluctuations.

### Neurophysiological and psychological findings

Additional studies explored neurophysiological and psychological domains alongside traditional cognitive outcomes. Ali et al. (2016) highlighted more frequent electroencephalogram (EEG) abnormalities and psychological impairments in PD patients than in HD. However, cognitive performance in visuospatial and executive tasks was marginally better among HD patients. These findings suggest potential baseline neurological vulnerabilities among PD patients, potentially influencing cognitive outcomes in specific domains.

### Critical appraisal

#### Risk of bias

**Fig. 7:**
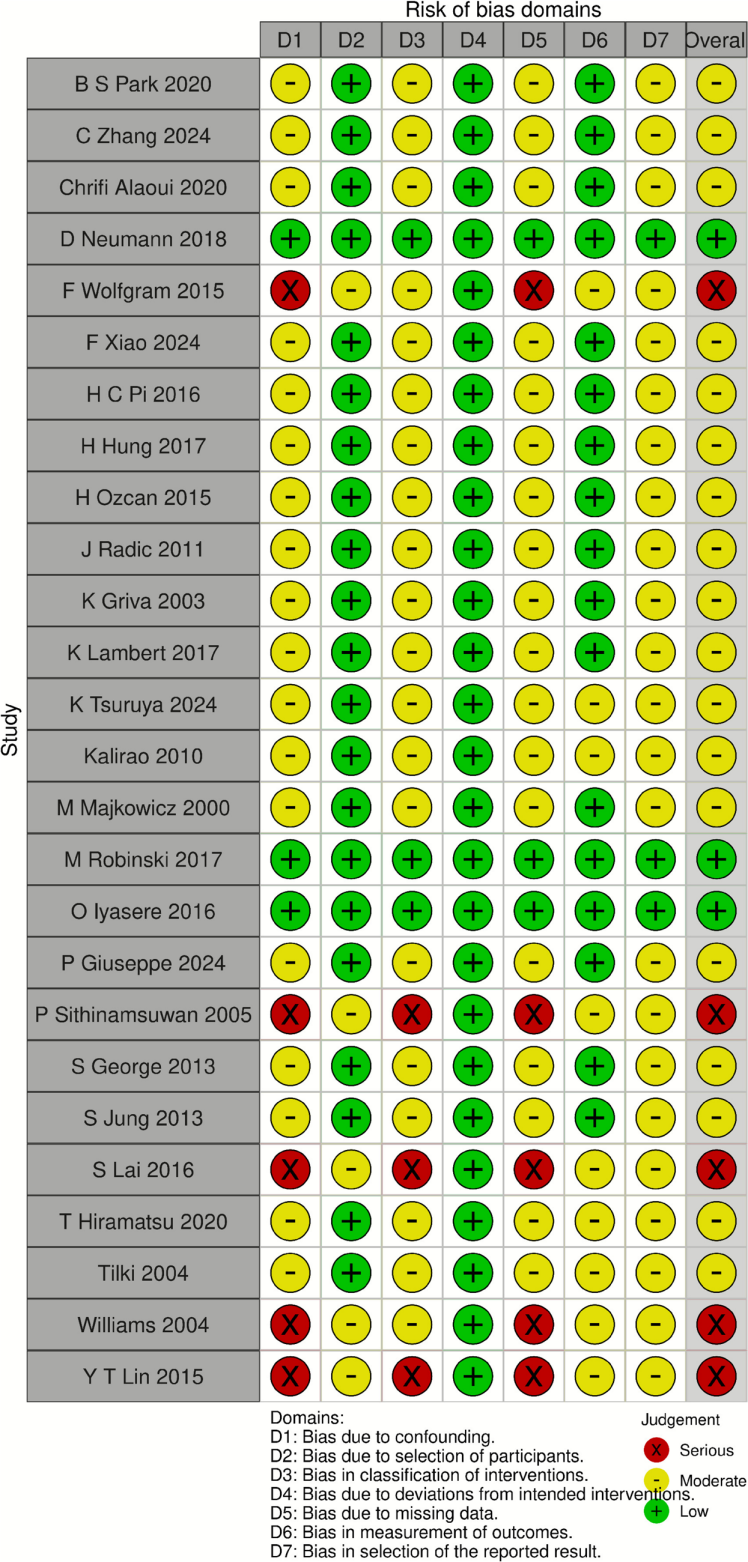
Summary of Risk of bias Analysis for non randomized cohort studies Deemed Suitable for Inclusion in This Review and Assessed Using the Cochrane ROBINS-I Tool (created using robvis tol)

Quality assessments conducted using the ROBINS-I tool indicated that 19 of the included studies exhibited moderate risk, three demonstrated low risk, and five presented serious risks of bias. The detailed results of the risk assessment are summarized in Fig. 7.

### Methodological quality

Assessment using the GRADE framework revealed that results from six studies warranted low confidence, two provided moderate confidence, and 19 studies offered high confidence in reported outcomes. These assessments are detailed in Table 3.

**Table 3:** Summary of Quality Appraisal of This Studies included in this Review Using the GRADE Tool

Study	Outcome	Risk of bias	Inconsistency	Indirectness	Imprecision	Publication bias	Overall Recommendation (Confidence in Outcomes)
B S Park et al	Brain connectivity (PD vs HD)	Moderate	Not serious	Not serious	Not serious	Not serious	High Confidence
C Zhang et al	rs-fMRI brain activity (PD vs HD)	Moderate	Not serious	Not serious	Not serious	Not serious	High Confidence
Chrif Alaoui et al	Quality of Life (PD vs HD)	Low	Not serious	Serious concerns	Serious concerns	Not serious	Low Confidence
D Neumann et al	Cognitive function (PD vs HD)	Low	Not serious	Not serious	Not serious	Not serious	High Confidence
F Wolfgram et al	Dementia risk (PD vs HD)	Serious	Some concerns	Not serious	Not serious	Not serious	Low Confidence
F Xiao et al	Gray matter changes (HD vs PD)	Moderate	Not serious	Not serious	Not serious	Not serious	High Confidence
H C Pi et al	CKD and cognitive impairment	Moderate	Not serious	Not serious	Not serious	Not serious	High Confidence
H Hung et al	Dementia risk in ESRD	Moderate	Not serious	Not serious	Not serious	Not serious	High Confidence
H Ozcan et al	Cognitive function, anxiety, depression	Moderate	Not serious	Not serious	Not serious	Not serious	High Confidence
J Radic et al	Motor/cognitive function HD vs PD	Moderate	Not serious	Not serious	Not serious	Not serious	High Confidence
K Griva et al	Neuropsych changes HD vs PD	Moderate	Not serious	Not serious	Not serious	Not serious	High Confidence
K Lambert et al	Cognition across CKD stages	Moderate	Not serious	Not serious	Not serious	Not serious	High Confidence
K Tsuruya et al	Brain atrophy progression (PD vs HD)	Moderate	Some concerns	Not serious	Not serious	Not serious	Moderate Confidence
Kalirao et al	Cognitive impairment in PD	Moderate	Not serious	Not serious	Not serious	Not serious	High Confidence
M Majkowicz et al	QoL in ESRD (HD vs PD)	Moderate	Serious Concerns	Not serious	Not serious	Not serious	Low Confidence
M Robinski et al	CKD and cognitive decline	Low	Not serious	Not serious	Not serious	Not serious	High Confidence
O Iyasere et al	Cognitive trends in advanced CKD	Low	Not serious	Not serious	Not serious	Not serious	High Confidence
P Giuseppe et al	Cognitive impairment across RRTs	Moderate	Some Concerns	Not serious	Not serious	Not serious	Moderate Confidence
P Sithinamsuwan et al	Neuro outcomes in HD/PD	Serious	Serious Concerns	Not serious	Not serious	Not serious	Low Confidence
S George et al	Cognition in dialysis with PFO	Low	Not serious	Not serious	Not serious	Not serious	High Confidence
S Jung et al	Cognitive variation in ESRD	Moderate	Not serious	Not serious	Not serious	Not serious	High Confidence
S Lai et al	Cognitive/psych outcomes in CKD therapies	Serious	Some concerns	Not serious	Not serious	Not serious	Low Confidence
T Hiramatsu et al	QoL and depression in HD vs PD	Moderate	Not serious	Not serious	Not serious	Not serious	High Confidence
Tilki et al	P300 EEG changes in HD vs PD	Moderate	Not serious	Not serious	Not serious	Not serious	High Confidence
Williams et al	Memory/attention post dialysis	Serious	Some concerns	Not serious	Not serious	Not serious	Low Confidence
Y T Lin et al	Dementia risk in HD vs PD (population-based)	Serious	Not serious	Not serious	Not serious	Not serious	Low Confidence

### AMSTAR 2.0 evaluation of previous reviews

Critical appraisal of previously published systematic reviews using the AMSTAR 2.0 tool identified limitations primarily related to incomplete search strategies, inadequate justification for study exclusion, failure to disclose funding sources, omission of risk-of-bias assessments, and insufficient examination of publication bias. Table 4 outlines specific item deficiencies identified in prior reviews, including this review, which explores every item. The main domains where deficiencies were identified include item 7 (justifications as to exclusion), item 10 (identification of sources of funding), item 12 (risk of bias discussion), and item 15 (publication bias discussion).

**Table 4:** Summary of Quality Appraisal of This Study and Older Systematic Reviews Using the AMSTAR-2.0 Tool

Author	Critical Flaws	Non Critical flaws	Overall Recommendation (Confidence in Outcomes)
Ali et al. 2021	3 (item 7, 13, 15)	2 (item 10, 12)	CRITICALLY LOW
Tian et al. 2019	3 (item 7, 13, 15)	2 (item 10, 12)	CRITICALLY LOW
Malik et al. 2025—This Review	0	0	HIGH

## Discussion

### Summary of main findings and significance

To the best of our knowledge, this systematic review and meta-analysis provides the most comprehensive and up-to-date synthesis of evidence regarding cognitive outcomes associated with HD and PD in patients with CKD. Our qualitative analyses suggest cognitive advantages favouring PD over HD, particularly within domains such as executive function, memory, processing speed, and long-term cognitive integrity (dementia incidence). The quantitative analyses revealed statistically significant overall differences between dialysis modalities.

Given the rising prevalence and substantial morbidity associated with CI in CKD patients [10], these findings hold considerable clinical importance. Enhanced understanding of how dialysis modalities differentially affect cognition could profoundly inform clinical decision-making, enabling a tailored approach to dialysis modality selection and improved quality of life for CKD patients.

Our findings are broadly consistent with two previous systematic reviews conducted on this subject. Similar to earlier reviews, our results identified trends toward PD-related cognitive benefits [15, 16]. However, methodological weaknesses limited prior reviews, including incomplete search strategies, variable quality appraisal methods, and outdated literature searches. By addressing these limitations through adherence to PRISMA guidelines, comprehensive risk-of-bias assessments, and thorough critical appraisal using the AMSTAR 2.0 checklist, our review offers a robust and timely update to the existing literature, significantly enhancing the reliability of its conclusions.

### Clinical implications

Clinically, our findings highlight potential cognitive benefits associated with PD, supporting its consideration, particularly among ESRD patients at elevated risk for cognitive decline. Continuous toxin clearance, stable haemodynamic profiles, and improved preservation of residual renal function with PD are potential mechanisms underpinning these cognitive benefits. Nevertheless, selecting dialysis modalities must remain individualised, carefully weighing cognitive considerations alongside patient-specific medical, psychosocial, and logistical factors.

### Impact of heterogeneity on findings

The findings of this meta-analysis should be interpreted with consideration for the observed statistical heterogeneity. Global cognitive function demonstrated moderate heterogeneity (I^2^ = 49%), which may reflect differences in the cognitive assessment tools employed, varying follow-up durations, or patient characteristics such as comorbidities. Subgroup analyses were employed to explore the root cause of heterogeneity; analyses of specific cognitive domains revealed that processing speed and memory both exhibited no heterogeneity (I^2^ = 0%), suggesting that these aspects of cognition are consistently affected across studies and may be more directly influenced by the physiological consequences of renal replacement therapy rather than study design variability.

By contrast, executive function still displayed moderate heterogeneity (I^2^ = 31%), which could be attributed to the broader and more complex nature of executive tasks, encompassing multiple cortical and subcortical networks that may be differentially affected by vascular, metabolic and uremic factors in HD and PD populations.

For dementia incidence, heterogeneity was high (I^2^ = 94%), indicating substantial variability across studies. This is likely due to differences in diagnostic criteria, follow-up duration, and population age structure. However, despite the statistical heterogeneity, the direction of effect was very consistent, showing a higher incidence of dementia in HD patients. This pattern may support the hypothesis that PD offers a relative protective effect on long term cognitive outcomes, possibly through greater haemodynamic stability, and lower cerebral microvascular injury compared with HD.

### Strengths

This systematic review exhibits several methodological strengths, enhancing the reliability and relevance of its findings. Firstly, our rigorous protocol included prospective registration (PROSPERO), adherence to PRISMA guidelines, comprehensive literature searches without language restrictions, and dual independent reviewers at all stages. The robust critical appraisal using ROBINS-I and GRADE frameworks provides transparent risk-of-bias and quality assessments, clarifying the strength of the evidence presented. Our extensive AMSTAR 2.0 evaluation of prior reviews further highlights our study's comparative methodological rigor. Additionally, including diverse international studies ensures the broad applicability of our findings across varied healthcare contexts, augmenting clinical relevance.

### Limitations

Despite its strengths, our review is constrained by some limitations. Most notably, the absence of RCTs within the literature means our evidence primarily derives from observational studies, which are inherently vulnerable to selection, confounding, and reporting biases. Variability in cognitive assessment methodologies and a lack of standardised neurocognitive protocols further limit comparability across studies. Differences in dialysis protocols, particularly duration and dosing schedules, complicate interpretations regarding optimal modality selection. Furthermore, most studies assessed short-term cognitive outcomes, potentially limiting insights into long-term cognitive trajectories in CKD patients.

Additionally, limited data exist regarding critical secondary outcomes such as patient-reported quality of life, depression, anxiety, and caregiver burden—all factors that could significantly influence cognitive outcomes and patient well-being but were beyond the scope of our review.

## Recommendations for Future Research

Future research should address identified limitations through high-quality longitudinal, multicentre, randomized controlled trials explicitly designed to evaluate the comparative cognitive impacts of HD and PD. Uniform application of standardised cognitive testing protocols and comprehensive neuroimaging would significantly enhance mechanistic understanding and clarify dialysis-related cognitive trajectories. Furthermore, investigations exploring long-term cognitive outcomes, secondary psychological outcomes, and patient-centered factors such as treatment autonomy, satisfaction, and overall quality of life will provide a more holistic evaluation of dialysis-related cognitive health.

## Conclusion

In conclusion, this systematic review and meta-analysis underscore the complex interplay between dialysis modality and ESRD patients' cognitive outcomes, suggesting PD's cognitive benefits relative to HD. Despite observational study designs limiting definitive conclusions, our findings support PD's cognitive advantages in specific domains. These insights have important implications for clinical practice, particularly for informed decision-making and patient counseling regarding dialysis modality choice. Rigid prospective studies incorporating comprehensive cognitive and patient-centered outcome measures are essential to optimize cognitive health and overall quality of life among ESRD patients undergoing dialysis.

## Supplementary Information

Below is the link to the electronic supplementary material.

Supplementary file1 (PDF 164 KB)

Supplementary file2 (PDF 118 KB)

Supplementary file3 (PDF 259 KB)

Supplementary file4 (PDF 243 KB)

## Data Availability

The data supporting this study’s findings are available from the corresponding author upon reasonable request.
