# Supplementary material for: Dialysis modality and cognitive outcomes in chronic kidney disease: a systematic review and meta-analysis
Source: Clin Exp Nephrol. 2025 Dec 1;30(3):408–23. doi: 10.1007/s10157-025-02798-2 (PMC12950099; doi:10.1007/s10157-025-02798-2)
Supplement: Supplementary file 2 — Supplementary file2 (PDF 118 KB) [file 10157_2025_2798_MOESM2_ESM.pdf]

Dialysis Modality and Cognitive Outcomes in Chronic Kidney Disease: A Systematic Review and Meta-Analysis

Clinical and experimental Nephrology

Ali Malik ; Hamid Reza Khademi Mansour ; Sukruth Pradeep Kundur ; Aryan Hunjan ; Rumail Zaheer.

Faculty of Life Sciences & Medicine, King's College London, London, United Kingdom
Email: ali.t.malik@kcl.ac.uk

SUPPLEMENT 2: SEARCH STRATEGY

Pubmed and PsycInfo	(chronic kidney disease OR CKD OR CKD [mesh] OR end-stage renal disease OR ESRD OR ESRD [mesh]) AND (cognit* OR cognition [mesh] OR executive function OR executive dysfunction OR dementia OR delirium OR amnestic OR mental status OR Neuropsych* OR memory) AND (hemodialysis) AND (peritoneal dialysis)
Ovid (Embase and Medline)	((chronic kidney disease or CKD).mp. or Renal Insufficiency, Chronic/ or end-stage renal disease.mp. or ESRD.mp. or Kidney Failure, Chronic/) and (cognit*.mp. or Cognition/ or executive function.mp. or executive dysfunction.mp. or dementia.mp. or delirium.mp. or amnestic.mp. or mental status.mp. or Neuropsych*.mp. or memory.mp.) and hemodialysis.mp. and peritoneal dialysis.mp. [mp=ti, ab, hw, tn, ot, dm, mf, dv, kf, fx, dq, bt, nm, ox, px, rx, an, ap, ui, sy, ux, mx]
Cochrane	(chronic kidney disease OR CKD OR end-stage renal disease OR ESRD) AND (cognit$ OR executive function OR executive dysfunction OR dementia OR delirium OR amnestic OR mental status OR Neuropsych$ OR memory) AND (hemodialysis) AND (peritoneal dialysis)
