# Supplementary material for: Dialysis modality and cognitive outcomes in chronic kidney disease: a systematic review and meta-analysis
Source: Clin Exp Nephrol. 2025 Dec 1;30(3):408–23. doi: 10.1007/s10157-025-02798-2 (PMC12950099; doi:10.1007/s10157-025-02798-2)
Supplement: Supplementary file 3 — Supplementary file3 (PDF 259 KB) [file 10157_2025_2798_MOESM3_ESM.pdf]

Dialysis Modality and Cognitive Outcomes in Chronic Kidney Disease: A Systematic Review and Meta-Analysis

Clinical and experimental Nephrology

Ali Malik ; Hamid Reza Khademi Mansour ; Sukruth Pradeep Kundur ; Aryan Hunjan ; Rumail Zaheer.

Faculty of Life Sciences & Medicine, King's College London, London, United Kingdom
Email: ali.t.malik@kcl.ac.uk

SUPPLEMENT 3: FULL STUDY CHARACTERISTICS INCLUDING STUDY DESIGN, LOCATION, AND SAMPLE SIZE

Study	Publication	Study Setting	Study Period	Study Type	Total	HD	PD	Follow up time	Main outcomes	Key findings	Funding
A C Aloui 2021	Wiley	Morocco, University hospital	October, 2018	Cross-sectional study	91	71	20	N/A	KDQOL SF v1.3, MCS	The univariate analysis showed that the Physical Component Score (PCS), the cognitive function, and the dialysis staff encouragement were better in PD patients (p = 0.03, p = 0.04, and p = 0.007, respectively), while the multiple linear regression indicates that the PCS, the dialysis Staff encouragement, and the patient's satisfaction subscores decreased within patients on	No funding

										HD compared with those on PD (p = 0.01, p = 0.03, and p = 0.02, respectively) and that the burden of the kidney disease and the work status subscores increased within patients on HD against those on PD (p = 0.007 and p = 0.04, respectively).	
BS Park 2020	Wiley Brain and Behaviour	Haeundae Paik Hospital, Inje University College of Medicine, Busan, Korea	October 2018 to March 2019	Cross-sectional study	40	20	20	N/A	VBM MRI, Consortium to Establish a Registry for Alzheimer's Disease Assessment Packet (CERAD-K), Verbal Fluency test, the Trail Making Test B, Stroop test color reading, the Korean version of the Boston	Significant cognitive decline in PD, no statistical difference in HD	2018 Inje University research grant

									Naming Test (K-BNT), 10-word list immediate memory, 15-min delayed recall and recognition, delayed figure recall, constructional praxis, and the Mini-Mental State Examination (MMSE) in the CERAD-K (MMSE-KC). The Verbal fluency test, the Trail Making Test B test, and the Stroop test		
C Zhang 2024	Cerebral Cortex	Conducted at various institutions in China,	May 2021 and May 2022	cross-sectional study	101 (49 healthy)	25	27	N/A	neuropsychological testing (MMSE, MoCA scores), rs-fMRI parameters such	significant alterations in brain activity patterns between the HD and PD groups. No significant difference in cognition	This study was supported by the Natural Science

		including the Medical School of Chinese PLA, First Medical Center of Chinese PLA General Hospital, and other affiliated centers.							as fractional amplitude of low-frequency fluctuations (fALFF), and Regional Homogeneity (ReHo)	between HD and PD	Foundation of China
D Neumann 2018	Kidney International	Germany	May 2014 - May 2015	Longitudinal study	271	163	108	1 year	Cognitive function (Trail Making Test-B, d2-Revision-Test, KDQOL-CF)	PD was associated with better cognitive function outcomes than HD; improvements observed in both groups over time.	German Federal Ministry of Education and Research
F Wolfgram 2015	Peritoneal Dialysis International	United States, using USRDS data	2006 - 2008	Retrospective cohort study	121, 623	112,960	8,663	Up to 3.75 years	Incidence of dementia in ESRD patients	PD patients had a lower incidence of dementia compared to HD patients (3.9% vs. 7.3% over 3 years); HD modality associated with higher dementia risk	Supported by the National Center for Advancing Translational Sciences, Nation

										even after adjustments.	al Institutes of Health (Grant Number 8UL1TR000055) and resources and facilities at the Clement J Zablocki VA Medical Center in Milwaukee, WI
F XIao 2024	Frontiers in Neuroscience	Conducted at various hospitals in China, including the First Medical Center of Chinese PLA General Hospital.	June 2021 to June 2022	Cross-sectional study	94 (42 healthy)	25	27	N/A	Brain gray matter volume changes in patients with CKD on hemodialysis (HD) and peritoneal dialysis (PD).Correlation of cognitive impairment scores (MMSE and MoCA)	Both HD and PD groups showed gray matter volume reductions compared to controls, with different affected brain regions.HD patients had more substantial volume reductions in some brain areas than PD patients.Cognitive function scores (MMSE and MoCA) were positively correlated	This study was funded by various grants from the Shanghai Committee of Science and Technology and the Shanghai Municipal Health

									with gray matter volume changes.	with gray matter volume.	Commission
HCPi 2016	Kidney Blood Pressure Research	Peking University First Hospital, China	March 2013 - March 2015	Cross-sectional study	120 (30 ND)	30	60	N/A	Cognitive function; Modified Mini-Mental State Examination (3MS), Trail-Making Tests A and B, Repeatable Battery for the Assessment of Neuropsychological Status (RBANS) neuroimaging (MRI: white matter hyperintensity, sulcal/ventricular atrophy)	HD had more severe neuroimaging abnormalities	Supported by New Century Excellent Talents from Education Department of China, Baxter Corp of China, ISN Research Award
HHung 2017	Oncotarget	Data from Taiwan's Longitudinal Health Insurance	January 1, 1999, to December 31, 2010	Retrospective cohort study	145, 868 (72,934 matched controls)	63,372	9,562	Average follow-up time was 4.24 years for HD patients	Incidence of dementia, including Alzheimer's disease (AD), vascular dementia (VaD),	HD patients: Adjusted Hazard Ratio (aHR) = 1.64, indicating a 64% higher risk of developing dementia compared to matched controls.PD	This study was funded in part by the National Cheng Kung University,

		Data base						and 3.23 years for PD patie nts.	and unspecifi ed dementi a (UnD).	patients: aHR = 2.21, indicating a 121% higher risk of developing dementia compared to matched controls.	Taiwa n Ministr y of Health and Welfar e Clinica l Trial and Resea rch Center of Excell ence, China Medic al Univer sity Hospit al, Acade mia Sinica Taiwa n Bioban k, Tseng-Lien Lin Found ation, Taiwa n Brain Diseas e Found ation, and the Katsuz o and Kiyo Aoshi ma Memor ial Funds

H Ozcan 2015	Transplantation Proceedings	Department of Nephrology, Ataturk University, Erzurum, Turkey	January to May 2014	Cross-sectional study	181 (69 KT)	54	58	N/A	Assessment of cognitive function using the Brief Cognitive State Examination (BCSE) Evaluation of anxiety and depression symptoms using the Hospital Anxiety and Depression Scale (HADS).	The cognitive impairment and mood disturbances were most severe in the HD group, moderate in the PD group, and mild in the KT group. KT patients scored significantly better on the BCSE than both HD and PD patients, indicating better cognitive function.HD patients exhibited the highest levels of anxiety and depression, followed by PD patients, with KT patients showing the lowest levels.	Not specified
J Radic 2011	Renal Failure (2011)	Conducted at the University Hospital Split, Croatia	Not specified	Cross-sectional study	42	22	20	N/A	Symbol Digit Modalities Test (SDMT) and Complex Reactiometer Drenovac (CRD-series).	No significant difference in cognitive and motor function between patients on HD and PD. Higher serum levels of albumin, creatinine, and calcium were associated with better cognitive and motor	N/A

										performance among all patients.	
K Griva 2003	Health Psychology	University College London and other hospitals in London, UK	Not specified	Cross-sectional study	145	77	68	Assessments conducted pre- and 24 hours post-dialysis (for HD)	Trail making test • Acute neuropsychological (NP) changes in ESRD patients undergoing HD and PD.	HD patients showed significant improvements in neuropsychological performance 24 hours post-dialysis across multiple cognitive domains, likely due to physiological recovery.PD patients showed minimal changes, reflecting the stability of continuous therapy.Changes in certain biochemical markers (urea, creatinine, potassium, etc.) aligned with cognitive improvements in HD patients, though biochemistry and NP improvements were not consistently correlated.	Funded by the Alexandros Onassis Foundation and the Reta Lila Weston Institute for Neurological Studies
K Lambert 2017	Nephrology (2017)	renal unit in large Australian	Not specified	cross-sectional study.	155	54	25	N/A	The study assessed cognitive impairment (CI)	Cognitive impairment was prevalent in patients with ESKD, especially in	The study was funded by the Australian

		Hospital							using the Montreal Cognitive Assessment (MoCA) tool.	those undergoing dialysis (HD and PD). HD patients had the highest prevalence of CI (55.6%), followed by PD (48%), with significantly lower rates in pre-dialysis and kidney transplant groups.	and New Zealand Society of Nephrology.
K Tsuruya 2024	Hypertension Research	Conducted in Japan, as part of the Observational Study on Cerebro- and Cardiovascular Complications in Chronic Kidney Disease (VCOHP Study)	December 2008 to December 2013	Cross-sectional and longitudinal observational study	107	34	73	2 years for longitudinal analysis	Brain atrophy progression as measured by changes in gray matter volume ratio (GMR) over time using MRI. Comparison of annual GMR decline between PD and HD patients.	In cross-sectional analysis, PD patients showed a significantly lower baseline GMR than HD patients.Over the 2-year follow-up, PD patients exhibited a faster decline in GMR compared to HD patients (−0.68 vs. −0.28 percentage points/year), even after	The study did not receive financial support from pharmaceutical companies or specific funding agencies

										adjusting for confounding factors. The findings indicate that brain atrophy progresses more rapidly in PD patients, which may increase the risk of cognitive impairment.	
Kalirao 2010	American Journal of Kidney Diseases, 2011.	two urban peritoneal dialysis units in Minneapolis and St. Paul, Minnesota	the first year of a 3-year longitudinal study ??	Cross-sectional study	490	338	51	N/A	Modified Mini-Mental State Examination (3MS)7 to test overall (global) cognitive function; the Hopkins Verbal Learning Test-Revised (HVLRT-R)8 to test verbal memory (12-word list); Color Trails 1 and 2 (a test similar to Halsted-	PD patients had more memory impairment but less impairment in executive function compared to HD patients. 66.7% of PD patients had moderate to severe cognitive impairment, similar to HD patients (73.4%).	Supported by the Minneapolis Medical Research Foundation and the National Institute on Aging.

									Reitan Trails A and B, but using alternative colors instead of numbers for Trail B)		
M Majkowicz 2000	The International Journal of Artificial Organs, Vol. 23, No. 7, 2000.	Department of Nephrology, Medical University of Gdansk, Poland.	April 1995 to March 1996.	Cross-sectional study.	87	65	22	N/A	EORTC QLQ-C30, Cantrill ladder, and HADS.	HD patients reported significantly lower quality of life across physical, social, cognitive, and general functioning compared to controls.	N/A
M Robinski 2017	Nephrology Dialysis Transplantation (2017)	Multicentre study conducted in 55 dialysis centers across Germany.	Data collected 6 to 24 months after the initiation of dialysis.	cross-sectional study	482 (PSM)	241	241	6 to 24 months after the initiation of dialysis.	Treatment satisfaction (TS), psychological and physical state, cognitive functioning trail making test, and shared decision-making (SDM),	PD patients showed higher treatment satisfaction, autonomy, and cognitive functioning compared to HD patients.	grant from the German Ministry for Education and Research

									summation of all these tests		
O Iyasere 2016	Clinical Kidney Journal in 2017	Imperial College Renal and Transplant Centre, Hammersmith Hospital, London, UK	November 2013 to October 2015.	prospective cohort study	102	41	25	4 months for up to 2 years.	ontreal Cognitive Assessment (MoCA) Macarthur Competency Assessment Tool (MacCAT-T).	Hemodialysis patients experienced a more rapid decline in executive function compared to peritoneal dialysis patients. Dialysis patients (HD and PD) exhibited faster declines in cognitive function compared to CKD patients.	partially funded by Baxter Health care
P Giuseppe 2024	Journal of Clinical Nephrology	Federico II University Hospital, Italy	January 2024	Cross-sectional study	99 (33 Healthy)	33	33	N/A	Cognitive function measured using the Cognitive Reserve Index Questionnaire (CRIq), which includes scores for education, working activity, and leisure time.	HD patients had significantly lower cognitive reserve scores (total CRI and subscores) than PD patients and controls.PD patients scored higher on cognitive function compared to HD patients, though lower than controls.The study suggests cognitive impairment may be more severe in HD	The study did not have external funding; no sponsors were involved in study design or data analysis

										patients due to factors like fluid shifts and inflammation.	
P Sithinamsuwan 2005	Journal of the Medical Association of Thailand	Phramongkutklao Hospital, Bangkok, Thailand.	September 2003 to August 2004.	Cross-sectional study	90	60	30	N/A	Thai Mental State Examination (TMSE). Thai Depression Inventory (TDI).	In HD patients, the prevalence of dementia was 8.3%, while it was 3.3% in the CAPD group. The prevalence of depression was 6.7% in both HD and CAPD groups.	N/A
S George 2013	Nephron clinical practice	Royal Sussex County Hospital, Brighton, UK	2011 - 2012	Pilot cohort study	80	59	21	12 months	digit symbol test (DST), FAS verbal fluency (FASVF), digit span backwards (DSB) and trails (B-A) (TBA)	PD patients showed a more rapid cognitive decline than those on HD	N/A
S jung 2013	Yonsei Med Journal	Hallym University Kangnam Sacred Heart Hospital	July-October 2009	cross-sectional study	56	29	27	N/A	The Korean version of the mini-mental state exam (K-MMSE) was applied to	The CPD group showed significantly higher K-MMSE score (27.8±2.9 vs. 26.1±3.1, p=0.010) and lower K-BDI score (12.0±8.4 vs. 20.2±10.4,	N/A

									screen the patient's cognitive function, while the Korean version of the Beck Depression Inventory (K-BDI) was used for depression screening	p=0.003) compared with the MHD group. The percentage of patients with depression symptoms was higher in the MHD group (51.7% vs. 18.5%)	
S Lai 2016	Medicine	University Hospital "Policlinico Umberto I," Sapienza University of Rome, Italy	December 2013 - March 2015	cross-sectional study	99	15	16	N/A	EEG patterns, cognitive function (NPZ5 tests), psychological assessments (MMPI-2 and SAT-P), and correlations with CKD-related variables	CKD patients showed significant cognitive and psychological impairments, notably on EEG, particularly PD and HD patients. Lower eGFR and higher inflammation markers (CRP) correlated with poorer outcomes. CKD and KT patients had persistent mental health issues despite treatment.	Not funded
T Hiramtsu	Therapeutic	Konan-Kosei Hospital,	Patients referred betw	Prospective, observation	75	45	30	24 months	SF-36 scores, MMSE scores,	Physical and social component scores of the SF-36	N/A

2020	Apheresis and Dialysis	Japan.	een October 2013 and December 2016, with a 24-month follow-up.	al study.					CES-D scores	significantly improved in PD patients at 24 months com- pared with those observed at baseline (42.8 vs. 39.4; P < 0.05 and 46.4 vs. 37.3; P < 0.05, respectively); how- ever, scores remained unchanged in HD patients. MMSE scores were significantly decreased at 12 and 24 months in HD patients (29.0 vs. 26.0, 25.0; P < 0.05), but remained unchanged in PD patients. Moreover, CES-D scores significantly worsened at 24months in HD patients (12.8 vs. 16.5), but remained unchanged in PD	
Tilki 2004	Upsala Journal of Medical	Departments of Neurology and Internal Medicine	Not mentioned	cross-sectional study	52	25	17	N/A	P300 cognitive potential obtained from auditory stimuli with the oddball paradig	P300 latency was longer in HD patients than in the control group and CAPD patients.	N/A

	Sciences	Ondokuz Mayıs University, Medical Faculty, Samsun, Turkey							m and the Mini-Mental State (MMS) examination		
Williams 2004	American Journal of Kidney Diseases	Department of Medicine and Neuropsychology Unit, United Health Services Hospitals, Johnson City, NY.	2002	cross-sectional study	30	20	10	N/A	Dodrill Stroop, Kaufman Brief Intelligence Test, Beck Depression Inventory-II.	CAPD patients showed overall stable performance on measures of attention and memory. However, hemodialysis patients showed significant changes (P < 0.05) in auditory memory for both immediate and delayed recall, with the greatest impairment occurring 67 hours postdialysis.	Not mentioned
Y T Lin 2015	Scientific reports	Taiwan Longitudinal Health Insur	January 1, 1998 to December	Retrospective cohort study	55,264	52,332	3292	N/A	Diagnosis of dementia	During the study period, 3775 patients were diagnosed with dementia in the HD group	This work was supported in part by grants from

		ance Data base	31, 2007							(177.5 per 10,000 person-years incidence rate) and 181 patients in the PD group (145.9 per 10,000 person-years incidence rate).	the intram ural grants from the Kaohsi ung Medic al Univer sity Hospit al
