# Supplementary material for: Dialysis modality and cognitive outcomes in chronic kidney disease: a systematic review and meta-analysis
Source: Clin Exp Nephrol. 2025 Dec 1;30(3):408–23. doi: 10.1007/s10157-025-02798-2 (PMC12950099; doi:10.1007/s10157-025-02798-2)
Supplement: Supplementary file 4 — Supplementary file4 (PDF 243 KB) [file 10157_2025_2798_MOESM4_ESM.pdf]

Dialysis Modality and Cognitive Outcomes in Chronic Kidney Disease: A Systematic Review and Meta-Analysis

Clinical and experimental Nephrology

Ali Malik ; Hamid Reza Khademi Mansour ; Sukruth Pradeep Kundur ; Aryan Hunjan ; Rumail Zaheer.

Faculty of Life Sciences & Medicine, King's College London, London, United Kingdom
Email: ali.t.malik@kcl.ac.uk

SUPPLEMENT 4: Expanded summary of the baseline characteristics of included patients in this reviews' included studies.

Study	Modality	Age (Median, IQR/SD)	Female (%)	Education Level	HBP (yes, %)	Diabetes (yes, %)	Smoking (yes, %)	Alcohol (yes, %)	Income (Low, %)	Work Status (Inactive, %)	Other Key Findings
A C Aloui 2021	HD	50.0 [35.5; 60.0]	52.1	Illeterate: 66.2%, Elementary education: 33.8%	42.30	9.9	16.9	2.8	88.7	84.5	
	PD	57.5 [42.0; 63.0]	40	Illeterate: 35%, Elementary education: 65%	65	10	20	5	100	85	
B S Park 2020	HD	64.85 (7.199)*	60	Years: 10.15 (4.545)*	-	-	-	-	-	-	Observed significant changes in global functional connectivity, higher cognitive impairment

	PD	60.95 (5.414)*	50	Years: 10.60 (3.633)*	-	-	-	-	-	-	Reduced global structural connectivity but preserved functional connectivity
C Zhang 2024	HD	61.00 ± 11.47	60	-	88	80	-	-	-	-	Cognitive impairment linked with decreased brain activity in specific regions.
	PD	54.63 ± 10.27	48.15	-	92.59	44.44	-	-	-	-	PD linked to wider brain activity alterations than HD.
D Neumann 2018	HD	57.0 (15.0)	27.6	Lower: 30.9%, Medium: 51.2%, High: 17.9%	-	-	-	-	-	77.20%	Matched sample analysis shows that PD patients had better cognitive function.
	PD	56.0 (14.7)	34.3	Lower: 24.1%, Medium: 41.7%, High: 34.3%	-	-	-	-	-	67.60%	
F Wolfgram 2015	HD	64.1± 14.4	45.5	-	86.3	50	6.6	-	-	17.4	HD patients had higher rates of dementia compared to PD patients over a 3-year period (7.3% cumulative incidence vs. 3.9% for PD).

	PD	62.4±15.9	44.9	-	85.8	48.9	6.8	-	-	17.5	PD patients showed lower cumulative incidence of dementia compared to HD over 3 years, even after adjusting for baseline characteristics.
F XIao 2024	HD	61.0 ± 11.47	40	-	88	80	-	Excluded (criteria)	-	-	Significant brain gray matter volume reduction, higher MoCA/MMSE correlation with damage in frontal/temporal regions.
	PD	54.63 ± 10.27	51.85	-	92.59	44.44	-	Excluded (criteria)	-	-	Gray matter changes less severe than HD.
H C Pi 2016	HD	56.5 ± 11.8	36.7	College or higher: 33.3%	-	33.3	-	-	-	-	Systolic BP: 131 ± 15.1. Higher prevalence of WMH, sulcal, and ventricular atrophy.
	PD	57.7 ± 7.8	55	College or higher: 26.7%	-	45	-	-	-	-	Systolic BP: 126.2 ± 14.4. Comparable cognitive function to HD.
H Hung 2017	HD	61.2 (13.9)	51.1	-	88.8	44.6	-	-	-	-	Adjusted hazard ratio for dementia = 1.64 (95% CI: 1.58–1.71)

	PD	52.9 (15.0)	53.6	-	89	37.3	-	-	-	-	
H Ozcan 2015	HD	51.1 ± 12.5	-	-	-	-	-	-	-	-	HD patients had higher anxiety and depression scores, lower cognitive function compared to PD and KT groups.
	PD	51.33 ± 14.4	-	-	-	-	-	-	-	-	PD patients showed better cognitive function than HD but worse than KT. Anxiety and depression scores were moderate.
	KT	50.19 ± 16.5	-	-	-	-	-	-	-	-	KT patients had the best cognitive function, lowest anxiety, and depression scores among the groups.
J Radic 2011	HD	49.59 ± 11.64	45.5	13.00 ± 2.02 years	-	-	-	-	-	-	Smoking: 5.00 ± 7.95. Albumin level positively correlated with cognitive performance.
	PD	51.10 ± 10.66	25	12.20 ± 2.33 years	-	-	-	-	-	-	Smoking: 6.75 ± 10.92. Albumin and creatinine levels positively correlated

											with cognitive performance.
K Grava 2003	HD	48.22 ± 14.92	42.9	12.26 ± 5.69	94.8	7.8	-	-	-	-	
	PD	52.26 ± 13.26	26.5	12.49 ± 5.11	88.2	27.9	-	-	-	-	Includes CAPD and APD modalities.
K Lambert 2017	PRE	70 (63–76)	54.20 %	<12 years: 54.2%	-	-	-	-	-	-	4 (16.7%) cognitively impaired
	PD	70 (63–81)	48.00 %	<12 years: 72.0%	-	35.00 %	-	-	-	-	12 (48.0%) cognitively impaired
	HD	72 (58–77)	33.30 %	<12 years: 63.0%	-	51.10 %	-	-	-	-	30 (55.6%) cognitively impaired
	KT	58.5 (49–66)	38.50 %	<12 years: 44.2%	-	28.60 %	-	-	-	-	10 (19.2%) cognitively impaired
K Tsuruya 2024	HD	64 [57–70]	26.40 %	Not Reported	Not Reported	35.30 %	11.7	38.2	-	-	Age- and sex-adjusted gray matter volume ratio (GMR) lower than PD; slower annual decline in GMR compared to PD
	PD	61 [53	34.20 %	Not Reported	Not Re	39.70 %	16.4	52.1	-	-	Rapid progression of brain

		– 68]			ported					atrophy compared to HD; faster annual decline in GMR
Kalirao 2010	PD	57.5 ± 14.8	33.3	<12 years: 46.0%	-	41.2	-	-	-	-	Moderate to severe cognitive impairment observed in 66.7%.
	HD	71.2 ± 9.5	45.9	<12 years: 54.7%	-	46.8	-	-	-	-	Moderate to severe cognitive impairment observed in 73.4%.
M Majkowicz 2000	HD										QoL worse in all areas compared to controls except emotional functioning
	PD										QoL similar to controls except personal/social functioning; better emotional functioning than HD
M Robinski 2017	HD (PSM)	59.8 ± 15.9	34.4	Lower: 19.5%, Medium: 61%, High: 19.5%	-	-	-	-	-	-	Patients preferred less autonomy-seeking and received more social support.
	PD (PSM)	58.8 ± 16.0	27.8	Lower: 25.7%, Medium:	-	-	-	-	-	-	Patients had better cognitive function and higher autonomy-

				51%, High: 23.2%							seeking behavior.
O Iyasere 2016	HD	68.9	29.3	<12 years: 28.9%	-	46.3	-	-	-	-	Cognitive function declines faster in HD patients; executive function declines more in HD compared to PD.
	PD	72.8	24	<12 years: 0%	-	44	-	-	-	-	Cognitive function better preserved in PD compared to HD; executive function also better preserved.
P Giuseppe 2024	HD	65.2 ± 3.4	42	Primary: 21%, Middle: 36%, Higher: 33%, Degree: 9%	-	18	33	-	-	-	Residual diuresis (ml): 335 ± 120
	PD	61 ± 5.2	45	Primary: 15%, Middle: 24%, Higher: 45%, Degree: 15%	-	9	24	-	-	-	Residual diuresis (ml): 1500 ± 260
P Sithinamsuw	HD	53.67 ±	45	9.98 ± 4.67 (years)	95	20	-	-	-	-	Prevalence of dementia: 8.3%,

an 2005		15.84									Depression: 6.7%
	PD	55.67 ± 14.18	30	11.13 ± 4.83 (years)	83.3	33.3	-	-	-	-	Prevalence of dementia: 3.3%, Depression: 6.7%
S George 2013	HD (PFO)	63.0 ± 11.5	41.7	-	50	16.7	-	-	-	-	HD patients with PFO showed slightly greater cognitive decline but not statistically significant
	HD (no PFO)	56.7 ± 16.4	28.6	-	45.7	28.6	-	-	-	-	
	PD (PFO)	70.0 ± 2.4	60	-	80	20	-	-	-	-	PD patients showed a greater rate of cognitive decline than HD patients
	PD (no PFO)	59.4± 15.2	31.8	-	31.8	68.1	-	-	-	-	
S jung 2013	HD	55.8 ± 8.7	55.2	0-11 years: 48.3%	-	41.4	20.7	-	-	-	Higher depressive symptoms, lower cognitive scores. SBP: 147.2±11.3
	PD	52.4 ± 11.6	48.1	0-11 years: 48.1%	-	33.3	22.2	-	-	-	Lower depressive symptoms, higher cognitive scores: SBP: 130.0±15.2

S Lai 2016	HD	53.0 ± 14.1	60	-	-	-	-	-	-	-	Higher cognitive decline observed compared to control group. SBP: 127.7 ± 17.8
	PD	67.0 ± 10.7	37.5	-	-	-	-	-	-	-	Higher EEG abnormalities compared to other groups. SBP: 136.7 ± 11
T Hiramtsu 2020	HD	66.6 ± 8.4	33.3	-	-	29.60%	-	-	-	4 patients retired due to dialysis	Significant cognitive decline, increased depression scores over 24 months. SBP: 148.4 ± 16.4
	PD	63.1 ± 11.0	28.9	-	-	28.90%	-	-	-	1 patient retired due to dialysis	Better cognitive function and emotional stability over 24 months. SBP: 149.0 ± 8.9
Tilki 2004	HD	37.3 ± 2.7	48	-	68%	-	-	-	-	-	Significant cognitive decline, longer P300 latency, lower MMS scores pre-dialysis
	PD	44.2 ± 3.9	58.8	-	65%	-	-	-	-	-	Better cognitive performance, shorter P300 latency, higher MMS scores compared to HD

Williams 2004	HD	54.6 ± 2.9	50	High school diploma: 50%, Some college: 40%, College degree: 5%, Grade <=12: 5%	20	30%	-	-	65%	None	Significant cognitive decline over the weekend, worse performance on memory and attention tasks 67 hours after last dialysis
	PD	45.1 ± 4.8	50	High school diploma: 30%, Some college: 40%, College degree: 10%, Grade <=12: 20%	20	50%	-	-	80%	None	Stable cognitive performance, better memory and attention compared to HD group at all time points
Y T Lin 2015	HD	Mean: 60.4 ± 10.3	52.3	-	58.20%	47.70%	-	0.60%	42.20%	-	Higher dementia incidence (17.75 per 1,000 person-years), increased risk of dementia over time
	PD	Mean: 59.7 ± 10.5	55.5	-	64.40%	41.40%	-	0.30%	40.40%	-	Lower dementia incidence (14.59 per 1,000 person-

											years), similar survival rates compared to HD group
Zeng 2022	HD	53.96 ± 12.99	38.3	Primary: 26.96%, Secondary: 64.35%, Higher: 8.70%	25.22	27.83	-	-	26.09	-	Better cognitive performance (MoCA: 27 [22-28]), higher HRQOL scores
	PD	58.35 ± 11.07	63.3	Primary: 50.00%, Secondary: 38.00%, Higher: 12.00%	27.33	37.33	-	-	7.33	-	Lower cognitive performance (MoCA: 15 [12-20]), lower HRQOL scores
